# Engaging boys as “Structured Allies” to prevent gender-based violence against girls: Results from the CARE Tipping Point Initiative in Nepal

**DOI:** 10.1371/journal.pone.0320014

**Published:** 2025-05-15

**Authors:** Kathryn M. Yount, Robert Durr, Suniti Neogy, Yuk Fai Cheong

**Affiliations:** 1 Hubert Department of Global Health, Rollins School of Public Health, Emory University, Atlanta, Georgia, United States of America; 2 CARE, United States of America, Atlanta, Georgia, United States of America; 3 Department of Psychology, Emory College of Arts and Sciences, Emory University, Atlanta, Georgia, United States of America; Northeastern University, UNITED STATES OF AMERICA

## Abstract

Global efforts to end violence against women and girls (VAWG) include engaging men and boys. The CARE Tipping Point Initiative in Nepal engaged men and boys as structured allies in girl-centered movement building to prevent VAWG, including sexual bullying and gender harassment (SBGH). We assessed whether this initiative in Kapilvastu and Rupandehi districts in Western Nepal affected adolescent boys’ reports of ever witnessing SBGH of a girl by another boy. Eligible boys were unmarried, 12–16 years, and not intending to migrate over the next 24 months. 1,154 (93% of) eligible, consenting boys across 54 randomly selected clusters participated at baseline, and 1,143 (99.0% of) baseline participants were followed. Outcomes were ever witnessed acts of non-contact (0–7; 0/1) and contact (0–7; 0/1) SBGH of a girl by another boy or male peer. Difference-in-difference (DID) regressions were estimated to assess the unadjusted and adjusted average treatment effects on these outcomes for participants assigned to the Tipping Point Program (TPP), Tipping Point Plus Program (TPP+), or control. Reports of ever witnessing acts of *non-contact* SBGH increased from 64% at baseline to 77% at follow-up due to increased reporting of ever witnessing “writing sexual messages…about a girl.” In adjusted models, boys in the TPP+ group ever witnessed 0.42 fewer acts of non-contact SBGH than did boys in the control group. Reports of ever witnessing acts of *contact* SBGH *implausibly declined* from 42% at baseline to 38% at follow-up, and this trend did not differ across study arm in adjusted models. Thus, compared to the control group, the TPP+ group may have reported smaller increases in ever witnessing acts of non-contact SBGH and more often concealed previously reported acts of non-contact SBGH ever witnessed. Future intervention studies should assess bystander motivation, self-efficacy, and behavior as direct measures of boys’ allyship to prevent SBGH and VAWG.

## Introduction

Global movements to end violence against women and girls (VAWG) include efforts to engage men and boys [1]. Across programs and approaches, this engagement typically involves education to foster awareness of VAWG, efforts to change norms of masculinity that normalize men’s VAWG, and skill-building to foster nonviolence and gender equity in families, peer networks, communities, and organizations [2–4]. Agreement is emerging among scholars, practitioners, and policy makers that ending VAWG requires the full participation of communities—including men and boys [5].

Despite this emerging consensus, engaging men and boys in antiviolence movements is fraught with complexities [1]. As an ally movement, engaging men and boys involves mobilizing a socially privileged group to support the dismantling of inequities and harmful social norms that have normalized violence by its own members [6–8]. This paradox raises questions about how to engage men and boys in ways that challenge rather than reinforce gendered power relations [8,9], that center rather than supplant women’s voices and leadership in antiviolence movements [10], and that attract and sustain men’s and boys’ engagement as authentic allies. Answers about how to engage individual men are emerging [8,11]. As men’s antiviolence groups gain momentum in global efforts to prevent VAWG, refined strategies are needed to engage men and boys at organizational and community levels in contextually salient ways. Ultimately, the allyship of men and boys to reduce VAWG requires the strategic negotiation of these complexities so that men engage effectively and sustainably as individuals and collectives over time [1].

This article presents findings from the CARE Tipping Point Initiative (TPI) in Nepal, which engaged men and boys as structured allies in girl-centered movement building to prevent child, early, and forced marriage (CEFM) and other forms of VAWG. Findings with respect to the impacts of the Tipping Point Program packages on CEFM are presented elsewhere [12]; this article focuses on changes in boys’ accounts of ever witnessing sexual bullying and gender harassment against girls in their communities that may be attributable to the Tipping Point program packages to which boys were assigned.

## Background

### Prevalence of violence against girls in South Asia and Nepal

Of the 12 million girls who marry before age 18 each year [13], about 30% of these marriages occur in South Asia [13]. In 2010, nearly 46% of women 20–24 years in South Asia reported being married before age 18, which translates into 24.4 million women in the region, and about 130 million girls projected to experience child marriage between 2010 and 2030 [14]. In recent years, rates of child marriage have declined in South Asia [13,15] from 63% in 1985 to 45% in 2010 [13]. This regional trend is dominated by declines in India, where rates of child marriage declined from 47% in 2005 to 27% in 2016 [16]. Still, the practice remains widespread and concentrated in certain regions and cultural groups, requiring focused and contextualized efforts to accelerate change.

Related forms of violence [17] against girls in South Asia include *sexual bullying* [18,19] and *gender harassment* [20], whereby child marriage is seen as a mechanism to ‘protect’ girls from the stigma and dishonor of these anticipated or disclosed experiences. The fragmented treatment by researchers of acts of *sexual bullying* and *gender harassment* may mask their interrelated nature and common underpinnings among young people, presenting an incomplete picture of peer victimization especially in the lives of adolescent girls [21–23]. In response, some researchers have combined (i) sexualized bullying or harassment, (ii) bullying or harassment about sexuality, and (iii) bullying or harassment about gender expression, as interrelated dimensions of *sexual bullying* [24] or *gendered harassment* [25], arguing that the performance and (re)enforcement of repressive gender and sexuality norms are underlying drivers of these interrelated experiences [24–28].

According to available estimates, rates of sexual bullying victimization among adolescent girls range from 28.5% to 54.5% [29], and rates of sexual harassment range widely, from 3% to 93% depending upon the sample and measure of sexual harassment used [30,31]. In South Asia, based on the global school-based student health survey of adolescents 11–18 years in Bangladesh (n = 2,989), 2014 and in Nepal (n = 6,529), 2015, the prevalence of at least 1 day of bullying victimization in the prior 30 days was 24.5% in Bangladesh and 50.9% in Nepal [19]. In Nepal, rates of bullying were slightly higher for girls than for boys (51.3% versus 48.7%) [19]. Similarly, in a 2018 study of secondary school girls in Nuwakot and Tanahun districts, Nepal, girls reported experiencing 3.12 episodes of bullying victimization in the prior week [18].

Notably, existing estimates may not be comparable across contexts due to differences in the samples and approaches to measurement, and national and regional trends in prevalence are lacking. Small-sample studies suggest that exposure to sexual bullying may be more prevalent among girls than boys, particularly among 12–14 year-old students in parts of Pakistan (28.5% versus 17.8%) [29] and secondary-school students in parts of Nepal (55% girls; 45% boys) [32] Such bullying is associated with a range of adverse outcomes, such as loneliness, anxiety, missing school, tobacco use, sleep loss, and attempted suicide [29,33,34]. In Nepal, compared to adolescents with no exposure to bullying in the prior 30 days, adolescents with any exposure experienced significantly higher adjusted odds of loneliness, sleeping difficulty, and suicidal ideation, plan, and attempt [19].

### Defining allyship among men and boys in the prevention of VAWG

Efforts to prevent VAWG—including sexual bullying and gender harassment—typically are grounded in socio-ecological frameworks [35,36] that extend Bronfenbrenner’s work [37] and identify nested levels of the human environment as having reciprocal (and often reinforcing) influences on VAWG. Socio-ecological models of prevention identify several levels at which men and boys can be engaged to prevent VAWG. At *the intrapersonal level*, men and boys can be engaged by strengthening individual knowledge and skills, changing personal attitudes, and improving mental well-being. At *the interpersonal level*, engagement might involve promoting education and mental well-being among parents, couples, and peers and building allyship among social networks that enable men and boys to intervene in the problematic behavior of male peers. At the *community level*, engaging men and boys might involve fostering community mobilization, and at *the institutional level*, engagement might entail educating providers and employees and changing institutional policies, cultures, and practices. At the *societal level*, men’s and boys’ engagement might involve building coalitions across networks, changing societal norms about gender and masculinities through social marketing and the media, and influencing changes in laws and policies [2,4,38–40].

Across these strategies, feminist-informed programs often aim to increase men’s and boys’ awareness about VAWG, to transform historically harmful norms about gender and masculinities [41–43], and thereby, to deepen their commitment to ending such violence—by being role models and proponents of respectful relationships in their personal networks [44] and/or by engaging formally in violence-prevention organizations [45]. Scholars and practitioners still recognize a basic tension inherent in engaging men and boys in antiviolence work—whereby the process of critically exploring dominant norms of masculinity and inviting men to reimagine strongly held beliefs about their own gender means shedding the privileges that accrue to them on the basis of their gender. Not surprisingly, a recent global review of 114 violence prevention plans across 14 countries revealed that engaging men and boys as primary prevention advocates remains in its formative stages [46].

### Barriers to engage men and boys in the prevention of VAWG

An emerging literature has identified several challenges to engaging individual men in VAWG prevention. On the one hand, some men may perceive such prevention efforts as antagonistic toward and blaming of men [1,2,8], as a “women’s issue” without relevance to men [47], or as embedded in a feminist agenda with which men feel disassociated, uncomfortable, or opposed [1]. On the other hand, many men see VAWG as an important problem and want to help, but may not know how to contribute [47], or lack the knowledge or skills to take an active stand against violence [48]. Moreover, some men who become visible antiviolence allies or who speak up about the disrespectful behavior of other men may experience skeptical, negative, or homophobic reactions from male peers [42]. Organizers of men’s anti-VAWG efforts also have faced barriers to sustain men’s commitment [11].

To address these challenges, men’s ally programs have developed strategies to engage men in positive ways and to address the many initial barriers to their participation [49]. These strategies include strengths-based outreach efforts in which men are approached as partners in prevention [42], a programmatic focus on conversations of importance to men, such as fatherhood and relationships [49], and engaging men in more and more structured dialogues that support reflection on ideas about positive, healthy masculinities in which notions of strength and nonviolence are integrated [11].

Importantly, outreach to individual men occurs in larger communities, organizations, and social environments, where supports and challenges to preventing VAWG coexist. These multifaceted contexts clarify the need to connect with individual men in nuanced ways that acknowledge coexisting obstacles in the settings where outreach is occurring. Empirically, VAWG prevention efforts seem to foster sustained change when they operate at multiple levels and engage individuals and their communities concurrently [4,50]. Historically, however, “tested” VAWG prevention programs typically have operated at the individual level [51,52], and as a result, have lacked an understanding of how the broader context conditions the process and outcomes of organizations that involve men and boys in VAWG prevention.

Results from randomized-controlled trials of violence-prevention programs with components to engage men and boys are notable. First, a two-armed, matched-pair, cluster-randomized controlled trial of the Engaging Men as Accountable Partners (EMAP) program conducted between 2016 and 2018 in eastern Democratic Republic of Congo engaged groups of men in 16 weekly 3-hour facilitated discussions on gender/power, VAWG, and allyship. At follow-up, compared to men in the control group, those in the EMAP group had significantly lower intentions of becoming violent, lower acceptance of wife beating, stronger beliefs that a woman has the right to refuse sex, and more egalitarian gender attitudes [53]. In Uganda, a randomized controlled trial of SASA!—a community-mobilization intervention designed to change community attitudes, norms, and behaviors that result in gender inequality, violence, and increased HIV vulnerability for women—showed more favorable outcomes among men in SASA! communities relative to men in control communities. These included relative reductions in the acceptability of a man’s use of violence against his partner, relative increases in the acceptability of a woman refusing sex, and relative reductions in prior-year concurrent sexual partners among non-polygynous men [54]. However, a review of other randomized controlled trials—including Stepping Stones! in South Africa and Coaching Men into Boys in the United States—showed limited to no significant, sustained impacts on men’s violent behavior toward women [55]. In the same review, evidence from quasi-experimental studies—including of Phaphama Men, The Male Norms Initiative, and Yaari Dosti—suggested reductions in various forms of partner violence against women; however, inferences were limited to self-selected samples and relatively short durations of follow-up, such that any sustained effects of programming on men’s behavior were unknown [55]. A more recent systematic review of randomized controlled trials of programs that included men’s engagement components showed frequent, significant favorable effects on gender-equitable attitudes, but effects on men’s partner violence against women were limited and mixed [56]. While these studies of adult men’s IPV against women are informative, their applicability to programming that seeks to engage boys as structured allies to reduce sexual bullying and gender harassment against girls is uncertain.

### Objectives of analysis

To address this gap, this analysis assessed whether and to what extent the CARE TPI, implemented in two districts in Nepal, affected adolescent boys’ reports of witnessing other boys or peers engaging in sexual bullying or gendered harassment of adolescent girls. The CARE TPI was novel in its explicit complementary programming with boys and men to engage them in support of girl-led movement-building to prevent child marriage and other forms of VAWG. Findings provide critical insights about the impacts of a girl-centered, community-based, social-norms change and movement-building program that was designed to engage boys and fathers as structured allies in girl-led strategies to end CEFM and these other ‘day-to-day’ forms of VAWG.

## Methods

### Study setting

The study setting was Kapilvastu and Rupandehi districts in Lumbini Province of Western Nepal. Human development in Lumbini province has been lower than the national average for decades [57]. In 2019, this provincial deficit was apparent in a life expectancy at birth that was lower than the national average by one year (68.8 versus 69.7), a slightly lower schooling attainment for adults 25 years or older (5.0 versus 5.2 grades nationally), and a nearly 25% lower income per capita (2086 versus 2748 nationally in 2011 purchasing power parity [PPP] United States Dollars [USD]) [58]. Overall, schooling attainment has increased in Lumbini province, but the slight gender gap in schooling attainment favoring men by 0.2 grades (4.9 for women versus 5.1 for men) is expected to increase to nearly 1.0 grade (12.2 for women versus 13.1 for men) due to faster expected increases in schooling attainment for boys than girls. Also, despite similar rates of labor force participation among women (70.0%) and men (72.1%) 15 years or older, men’s per capita income is almost double that for women (2751 versus 1488 in 2011 PPP USD). On more direct measures of women’s agency, Lumbini Province ranks fifth among Nepal’s seven provinces in women’s capacity to participate in household decisions, with 34.4% of married women 15–49 years reporting that they participate in no household decisions [59]. Moreover, like six of seven provinces in Nepal, more than one in four women (27.7%) 15–49 years agree that wife-beating is justified for at least one reason [59]. In this context, the median age at first marriage for women 25–49 years is 17.7 years, the third lowest across provinces in Nepal and below the legal age at marriage of 20 years [59].

### Overview of the CARE Tipping Point Initiative in Nepal

**Fig 1:**
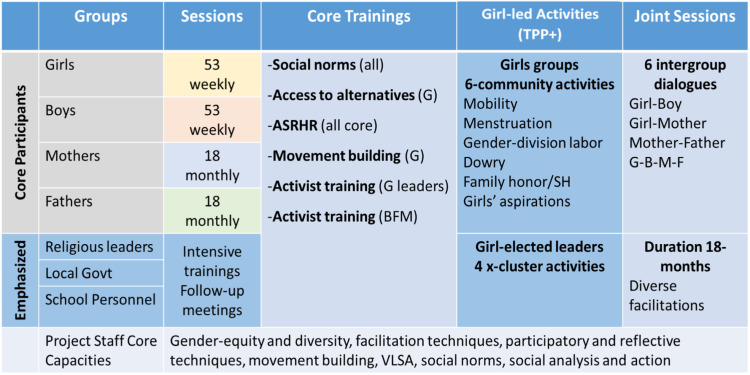
Planned CARE Tipping Point Program (TPP) and Tipping Point Program plus (TPP **+) packages.** Notes. ASRHR = Adolescent sexual and reproductive health and rights; G = Girl; BFM = Boy-Father-Mother; SH = Sexual Health; VSLA = Village Savings and Loan Association.

**Fig 2:**
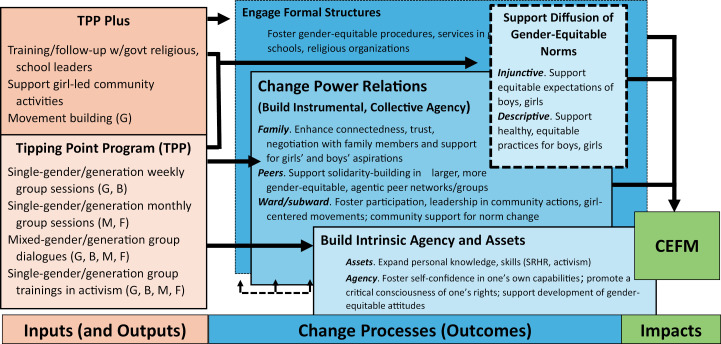
CARE Tipping Point Initiative: theory of change. Notes. Included with permission from Yount et al. [12].

CARE’s TPI in Kapilvastu and Rupandehi districts focused on addressing the underlying causes of CEFM, many of which are drivers of VAWG more generally. These drivers included poverty, gender inequality, and restrictive gender and social norms [60,61]. To address these drivers, CARE’s TPI in Nepal aimed to promote the rights of adolescent girls through community-level programming that involved the synchronized engagement of different participant groups to challenge social expectations and repressive gender norms and to promote girl-centric and girl-led activism (Fig 1). CARE’s TPI included a ‘core’ program package, the Tipping Point Program (TPP), with components to enhance adolescent girls’ *personal assets and intrinsic agency*, including their self-efficacy, as well as girls’ *instrumental agency*, including their voice and negotiation skills. CARE’s TPI also included an ‘enhanced’ program package, TPP + , with all components in the TPP as well as activities to enhance social-norms change by engaging activist boys, parents, and community leaders and by facilitating girl-led community activities and social-norms-change events. This intervention study, thus, was novel in its explicit design to disentangle the effects of the TPP and TPP+ packages on primary and secondary outcomes that mapped onto a refined theory of change. Fig 1 summarizes the planned components of the CARE TPP and TPP+ packages, and Fig 2 summarizes their theorized pathways of effect. Details are available elsewhere [62].

Programmatic modifications resulting from the COVID-19-induced lockdown and associated disruptions were recorded in the trial registry (https://www.clinicaltrials.gov/ct2/show/NCT04015856). In brief, the TPP and TPP+ packages were reduced in duration from 18 to 16 months (July, 2019 to March, 2021). A five-month hiatus in programming occurred from March, 2020 to July, 2020, and the number of weekly sessions for girls and boys were reduced from 45 to 38. To adhere maximally to the original programmatic content, some sessions were merged, and repetitive content was eliminated. In the TPP+ package, the enhanced community-level social-norms-change activities were conducted in a similarly consolidated manner. Originally, to capture sustained changes in primary and secondary outcomes beyond the intensive period of program implementation, a freeze period of 12 months was planned from the end of program implementation to follow-up data collection; however, due to COVID-19-related delays in the completion of program implementation, this freeze period was reduced to eight months.

### Tipping Point theory of change

The Tipping Point Theory of Change identified core input, change processes or intermediate outcomes, and impacts. *Inputs* included the core CARE TPP package and additional components of the CARE TPP+ package [12,62] (Fig 2). Core sessions for boys focused on norm change, adolescent sexual and reproductive health and rights, and structured activist training to become allies who support girl-led movement building (Fig 2). *Change processes* or intermediate outcomes were the mechanisms by which TPP and TPP+ were expected to operate. TPP+ sessions with community leaders, including school personnel, were expected to engage local formal structures to foster gender-equitable procedures and services and to facilitate shifts in gender norms among key reference groups. TPP and TPP+ were expected to change interpersonal power relations by increasing girls’ agency; solidarity and movement building among peers; and participation among community members—including boys—to support norms change. TPP and TPP+ were expected to support the diffusion of gender-equitable norms regarding what community members believed girls should do (injunctive norms) and actually do (descriptive norms). The facilitated diffusion of gender-equitable norms was emphasized in TPP + , through girl-centered movement building and its four related girl-led community-level activities, social-norms activities, engagement of key constituents, and inter-group dialogues. These intermediate normative and agency-related outcomes were expected to support a decline in CEFM, and intermediate outcomes and impacts were expected to be more pronounced in the group receiving TPP + . *Impacts* were the expected impacts of TPP and TPP + on girls’ risks of CEFM. Thus, the primary outcome of the project was to reduce CEFM among girls, and key intermediate outcomes were more gender equitable norms and greater allyship among boys.

### CARE strategy to engage fathers and boys to prevent CEFM and VAWG

With respect to engaging men and boys in the TPI, CARE found that formal and informal support structures were crucial to the success of young activist girls [63]. Therefore, CARE took the novel approach of seeking to strengthen the supportive capacities of boys and men around the girl as a means to ensure that this surrounding network would come alongside her as allies instead of as protectors, and as supporters instead of as guides. Members of existing boys’, fathers’, and mothers’ groups were offered the opportunity to challenge inequitable gender norms and to stand by the girls in their activism. These boys, fathers, and mothers were trained in separate workshops on how to be an ally to girls in their communities—specifically around girl-led activist activities (Fig 1). The ally training for men and boys (boys’, fathers’, and religious leaders’ groups) included sessions from SASA! and SteppingStones! that were adapted for Nepal. The training began with a discussion of their role as allies and the importance of an environment where girls felt free to question, raise their voices collectively, and take risks to advance social change at the community level. The sessions for this training included a reflection on whether actions taken were those of an ally or a protector in each situation. For instance, when a girl’s voice or opinion was ignored by those to whom she was speaking, and her brother restated her point or question, was he acting as an ally to lift and center her voice or was he centering his own voice in this space? The boys, fathers, and mothers also practiced beginning activist conversations with family members, spouses, friends, and neighbors. Field staff facilitated these sessions and coordinated with the mentors assigned to girl activists.

After the training, the boy, father, and mother allies met with the girl activists, who shared how they could be supported in their next public-facing, social-norm-shifting activity. After each such activity, the allies also engaged in review and reflection with the girl activists. The girls shared their experience and feedback on how they felt the sessions were managed and sought their feedback on how they felt the event was able to challenge the focal norm of the event. They also shared plans for the next event, asking for support in its execution or plan for risk mitigation. Thus, men and boys were trained to engage as structured allies who centered girls’ voices and activism at the community-level to change repressive gender norms tied to CEFM and VAWG, more generally.

### Ethical considerations

The Emory University IRB (IRB00109419) and Nepal local ethics committee (#161 2019) reviewed and approved the original protocol. No continuing IRB review was required; however, due to programmatic and design changes resulting from the COVID-19 pandemic, updated protocols were submitted and approved. Census households received a written consent detailing the study background, purpose, risks and benefits, confidentiality, and right to withdraw. In consenting households, oral informed consent of the parent and oral informed assent of the child were obtained before initiating any interview.

### Boys’ sample: Eligibility, participation, and retention

The full protocol for sample eligibility, participation, and retention of all participants in the parent study is detailed elsewhere [62]. Eligible boys for the Tipping Point intervention and data collection were unmarried, 12–16 years, and living in selected clusters at baseline, with no plans to migrate in the subsequent 24 months. To identify the sample, wards (the lowest governmental administrative unit in Nepal) were combined or segmented to create primary sampling units, or clusters, of roughly 200 households. From this sampling frame, 27 clusters were selected each from Kapilvastu and Rupandehi districts with probability proportional to size, for a total of 54 clusters. Clusters were assigned randomly to control, TPP, and TPP+ study arms, for a total of 18 clusters per arm. The likelihood of spillover between treated and control clusters was reduced because of the high geographic dispersion of households within clusters and the cluster-randomization process, which resulted in a substantial percentage of clusters being non-contiguous (See maps in Yount et al. [62] for more detail.).

Enumerators mapped and conducted a pre-baseline household census in each selected cluster. Census data were used to generate lists of eligible adolescent boys. For adequate power in the parent trial [62], 23 eligible boys were enrolled at baseline in each cluster, for a total expected sample size of 1,242 boys across clusters. Baseline interviews were completed with 1,154 eligible and assenting boys across all 54 clusters, a 92.9% baseline participation rate. Follow-up interviews were completed with 1,143 boys who had participated at baseline, a 99.0% retention rate.

### Data collection

In the baseline and follow-up surveys, the boys’ sample was administered 16 modules with questions that captured five constructs related to personal assets and intrinsic agency, instrumental agency, collective agency, social networks and norms, and discrimination and violence as a barrier to change [62]. All questionnaire modules were translated into Nepali and Awadhi, a primary dialect spoken in the districts. All translated questionnaires underwent cognitive testing, pre-testing, and pilot testing in the areas where the study was conducted, and questionnaires were revised to ensure that lay understandings of questions, terms, and responses aligned with their intended meaning in English. Interviewers were recruited from the local study areas so that respondents could be interviewed in their primary spoken language. Baseline data collection took place from June 10 to July 19, 2019, and follow-up data collection took place during December, 2021 and January, 2022.

#### Outcome measures.

Seven items captured *ever witnessing non-contact acts of bullying or harassment against a girl by another boy or male peer*. Each item was scored 0 (no) or 1 (yes) and was summed to have a theoretical range of 0–7, with higher scores indicating ever witnessing more acts. An example item was “spreading sexual rumors about a girl.” Alpha reliabilities were 0.68 at baseline and 0.59 at follow-up. Seven items captured *ever witnessing contact acts of bullying or harassment against a girl by another boy or male peer*. Each item was scored 0 (no) or 1 (yes) and was summed to have a theoretical range of 0–7, with higher scores indicating ever witnessing more acts. An example item was “forcing a girl to do something sexual other than kissing.” Alpha reliabilities were 0.71 at baseline and 0.63 at follow-up. All 14 items captured *ever witnessing any non-contact or contact acts of bullying or harassment against a girl by another boy or male peer*. Items, scored 0–1, were summed to have a theoretical range of 0–14, with higher scores denoting ever witnessing more acts. Alpha reliabilities were 0.79 at baseline and 0.75 at follow-up.

The choice to focus on boys’ reports of ever witnessing sexual bullying or gender harassment was made to reduce social desirability bias, as boys would not be reporting on their own behavior but instead would be reporting on observations of the behavior of other (unnamed) boys [64]. Also, given the cumulative or lifetime nature of the outcome and the panel study design, favorable negative programmatic impacts would be observed as *stable or more modest increases in reports of ever witnessing a boy or other male peer commit acts of bullying or harassment against a girl in the TPP and TPP+ groups compared to the control group*.

#### Individual-level covariates.

Individual-level covariates, selected from the team’s empirical research in the study population [12], included the adolescent boy’s baseline age in completed years, grades of schooling, literacy (could read and/or write, neither [reference]), vocational training (yes, no [reference]), religion (Hinduism, Muslim, and other [reference]), caste (advantaged, disadvantaged [reference]), household poverty score, main occupation of the household head (longterm employee, no job/doesn’t work [reference]), and participation of the boy or family member in another empowerment program in the prior two years (yes, no [reference]). Household poverty was measured using eight items from the Nepal 2010 Poverty Probability Index (PPI). These items had varying scores that were summed to have a possible range of 0–61. Questions included the male head’s/spouse’s job type worked the most hours in the past seven days, number of bedrooms in the residence, main construction material of outside walls and roof, in-home amenities (type of stove mainly used for cooking; type of toilet used; number of telephone sets/cordless/mobile owned), household ownership of agricultural land (own, sharecrop-in, mortgage-in) and, if yes, whether irrigated. This scale was inversely proportional, such that a higher score represented a lower likelihood of poverty and a score of twenty-five or below indicated a high likelihood of poverty.

#### Community-level covariates.

Community-level covariates included the baseline cluster-level proportion of households from an advantaged caste, proportion of households being Muslim, average household Poverty Probability Index (PPI), mean grades of schooling completed for women 25 years or older, and the gender gap in mean grades completed for adults 25 years or older (men’s mean grades – women’s mean grades) [60]. These measures were computed using data from the pre-baseline household census in each study cluster, so measures reflected the average for the population of households or adults 25 years or older in the cluster. A final cluster-level control measure was gender norms (hereafter “norms” for parsimony). Norms were measured by computing the cluster-level mean summative score for responses to 16 items among women and men 25 years or older who participated in the survey in each cluster [60,62]. Each item was coded 0 (fully agree) to 3 (fully disagree), or reverse coded to ensure a more gender-equitable valence. An example item was “most people in my village will approve if a married woman goes out of house to work.” Alpha reliabilities at the individual-level were 0.92 for women, 0.86 for men, and 0.91 for all adults. Items were summed for each adult, and summative scores were averaged in each cluster to capture the cluster (community-level) mean gender norm, with higher means denoting more equitable gender norms among adults in the cluster.

### Statistical analysis

#### Descriptive analyses.

As a first step in the descriptive analysis, individual items for each outcome were organized into conceptual item sets. Missing responses were coded as missing for univariate analyses of items and as 0 for summative scoring. Spearman pairwise correlations were estimated to ensure that items within sets were mutually correlated and that summative scales were reasonable reflections of intended outcomes (results available on request). An item was considered for deletion if the magnitude of its pairwise correlation with other items in the same set was close to zero and not significant. Scale reliabilities were assessed for all outcomes using Cronbach’s alpha for each item set or subset after item deletion.

As a second step in the descriptive analysis, we estimated univariate distributions for each item and for derived outcomes, individual-level covariates, and community-level covariates, for the total sample of boys and for boys within each study arm. Given the voluntary nature of participation of eligible participants in the clusters that were assigned randomly to TPP and TPP + , this step followed the published study protocol [62] to assess the extent of within-sample balance of outcomes and observed covariates at baseline across study arms. This step was performed and reported for the girls’ sample in the primary impact analyses [12] and enabled us to identify potential confounders of the impact of TPP and TPP + on primary and secondary outcomes, reported elsewhere for girls [12] and here for boys.

#### Assessment of program impact.

As a first step in the impact assessment, we estimated the average treatment effect on outcomes by computing the differences between the means of the outcomes for participants assigned to TPP or TPP+ versus non-participants, or those assigned to the control group. We used the difference-in-difference (DID) regression approach with cluster-robust variance estimators, which aims to eliminate the confounding effects of unobserved study-arm and time characteristics [65,66]. We first ran DID models without covariates. Then, we re-estimated all DID models, adjusting for the individual- and community-level covariates, described above. We assessed and compared the unadjusted and adjusted impacts of random assignment to the TPP treatment group or to the TPP+ treatment group, each relative to the control group. The two DID models took the following general forms. Let us denote the groups as indicator variables G_1_ for the control group, G_2_ for the TPP treatment group, and G_3_ for the TPP+ treatment group. The equation without covariates is:


$${\text{Y}}_{\text{it}}\;\text{=}\;{\;\;\;\beta \;\;\;}_{\text{0}}\;\text{+}\;{\;\;\;\beta \;\;\;}_{\text{1}}\text{Pos}{\text{t}}_{\text{t}}\;\text{+}\;{\;\;\;\beta \;\;\;}_{\text{2}}{\text{G}}_{\text{2}}\;\text{+}\;{\;\;\;\beta \;\;\;}_{\text{3}}{\text{G}}_{\text{3}}\;\text{+}\;{\;\;\;\beta \;\;\;}_{\text{4}}\left(\text{Pos}{\text{t}}_{\text{t}}\;\;\;\;\times \;\;\;\;{\text{G}}_{\text{2}}\right)\;\text{+}\;{\;\;\;\beta \;\;\;}_{\text{5}}\left(\text{Pos}{\text{t}}_{\text{t}}\;\;\;\;\times \;\;\;\;{\text{G}}_{\text{3}}\right)\;\text{+}\;\epsilon_{\text{it}}$$

where

β_0_ is the intercept, representing the baseline outcome for the control group in the pre-treatment periods;

β_1_ captures the time effect common to all groups;

β_2_, β_3_ represent the baseline differences between G_1_ (the control group) and G_2_ and G_3_, respectively;

β_4_, β_5_ denote the treatment effect for group G_2_ and G_3_, relative to G_1_, respectively; and

∊_it_ refer to individual-level deviations.

We included covariates (**X’**_it_) to control for individual- and community-level covariates:


$${\text{Y}}_{\text{it}}\;\text{=}\;{\;\;\;\beta \;\;\;}_{\text{0}}\;\text{+}\;{\;\;\;\beta \;\;\;}_{\text{1}}\text{Pos}{\text{t}}_{\text{t}}\;\text{+}\;{\;\;\;\beta \;\;\;}_{\text{2}}{\text{G}}_{\text{2}}\;\text{+}\;{\;\;\;\beta \;\;\;}_{\text{3}}{\text{G}}_{\text{3}}\;\text{+}\;{\;\;\;\beta \;\;\;}_{\text{4}}\left(\text{Pos}{\text{t}}_{\text{t}}\;\;\;\;\times \;\;\;\;{\text{G}}_{\text{2}}\right)\;\text{+}\;{\;\;\;\beta \;\;\;}_{\text{5}}\left(\text{Pos}{\text{t}}_{\text{t}}\;\;\;\;\times \;\;\;\;{\text{G}}_{\text{3}}\right)\;\text{+}\;{\text{X }}_{\prime }^{\text{i}t\gamma }\;\;\;\;\text{+}\;\varepsilon_{\text{it}}$$

where

**X**’_it_ denotes a vector of covariates; and

**γ** refers to a vector of coefficients associated with the covariates.

## Results

### Characteristics of the boys’ sample

On average, boys in the study districts were 14 years old and could read or write (94%) at baseline (Table 1). Almost all boys (94%) had ever attended school, and a majority (65%) had attended a government school, completing grade 6, on average. Most boys (85%) still were attending school at baseline, while very few had received vocational training (4%). Most boys (89%) were from Hindu households, and almost half were members of upper-caste groups (47%) or were relatively advantaged indigenous peoples (26%). Almost all (80%) male heads of household (or their spouse) worked in daily wage labor or self-employment as their main occupation in the seven days before baseline. During the two years before follow-up, only 4% boys and their families had participated in other empowerment organizations unaffiliated with TPI, and a majority of the sample had never participated in other empowerment organizations (67%).

**Table 1:** Characteristics of unmarried boys 12-16 years at baseline who were retained at follow-up, Kapilvastu and Rupandehi Districts, Nepal, 2019, overall and by Tipping Point Evaluation Study Arm (N = 1,143).

	Control (n = 391)	TPP (n = 390)	TPP+ (n = 362)	Total (n = 1,143)
Age in years, M (SE)	13.93 (0.07)	13.85 (0.07)	13.79 (0.07)	13.86 (0.04)
Can read or write, %				
Neither	2.05	4.87	2.49	3.15
Read and/or write	94.12	92.05	95.86	93.96
Missing	3.84	3.08	1.66	2.89
**Ever attended school, %**				
Yes	95.40	94.10	93.37	94.31
No	4.60	5.90	6.35	5.60
Missing	0.00	0.00	0.28	0.09
**School type attended, %**				
Government	63.94	66.41	66.30	65.53
Private	30.69	26.15	26.24	27.73
Community	0.77	1.28	0.83	0.96
Other	0.00	0.26	0.00	0.09
Never attended	4.60	5.90	6.35	5.60
Missing	0.00	0.00	0.28	0.09
Grades completed, M (SE)^1^	6.24 (0.12)	5.54 (0.13)	5.75 (0.13)	5.85 (0.07)
Still attending school, %				
Yes	84.14	86.41	83.15	84.60
No	11.25	7.69	10.22	9.71
Never attended	4.60	5.90	6.35	5.60
Missing	0.00	0.00	0.28	0.09
Ever received vocational training, %				
Yes	4.35	3.08	4.70	4.02
No	95.65	96.92	95.30	95.98
Missing	0.00	0.00	0.00	0.00
Religion, %				
Hinduism	91.82	87.44	86.19	88.54
Buddhism	0.26	0.26	0.28	0.26
Islam	6.91	12.31	12.71	10.59
Kirat	0.00	0.00	0.00	0.00
Christianity	0.51	0.00	0.83	0.44
Other	0.00	0.00	0.00	0.00
Don’t know	0.26	0.00	0.00	0.09
Missing	0.26	0.00	0.00	0.09
Caste, %				
Upper-caste groups	43.99	56.15	40.06	46.89
Relatively advantaged indigenous people	32.99	14.10	30.11	25.63
Disadvantaged Terai and religious minority groups	6.91	11.79	11.05	9.89
Disadvantaged indigenous groups	1.02	1.54	1.93	1.49
Dalit groups	14.07	15.38	14.36	14.61
Other	1.02	1.03	2.49	1.49
Missing	0.00	0.00	0.00	0.00
Household PPI, M (SE) [Theoretical range 0–61]	41.07 (0.54)	42.05 (0.50)	41.54 (0.55)	41.55 (0.31)
Male head/spouse primary occupation prior seven days, %				
No male	9.97	6.41	8.84	8.40
Does not work	2.81	3.08	2.76	2.89
Paid daily in agriculture	26.34	26.67	26.52	26.51
Paid daily in non-agriculture	13.81	15.38	13.81	14.35
Self-employed in agriculture	23.79	23.08	23.76	23.53
Self-employed in non-agriculture	15.86	15.90	14.64	15.49
Paid wages on a long-term basis in agriculture or non-agriculture	7.16	9.49	9.12	8.57
Missing	0.26	0.00	0.55	0.26
Participation in other empowerment organizations not affiliated with Tipping Point, %				
Yes, me and my family	0.26	0.77	2.49	1.14
Yes, me only	1.79	2.05	2.49	2.10
Yes, family only	1.79	0.77	0.55	1.05
No	69.57	66.92	64.36	67.02
Don’t know	11.25	16.15	14.36	13.91
Missing	15.35	13.33	15.75	14.79

^1^Three observations on grades of schooling were missing.

### Trends in ever witnessing sexual bullying or gender harassment

At baseline, nearly two thirds (64%) of boys reported ever witnessing another boy or male peer engaging in *non-contact* forms of bullying or harassment of girls (Table 2). The baseline reported prevalence of ever witnessing specific acts ranged from 8% for “flashing or ‘mooning’ a girl” to more than 50% for “making sexual comments…at any girl.” The reported prevalence of ever witnessing another boy or male peer “making sexual comments” remained stable at follow-up (52%), and the reported prevalence of ever witnessing another boy or male peer “writing sexual messages or graffiti (e.g., on bathroom walls, in locker rooms, in a note or book) about a girl” doubled from 25% at baseline to 50% at follow-up. However, the reported prevalence of ever witnessing all other listed acts of non-contact bullying or harassment *implausibly declined* from baseline to follow-up (Table 2). Overall, given the marked increase in reports of ever witnessing other boys write sexual messages, the reported prevalence of ever witnessing any non-contact bullying or harassment of a girl increased, from 64% at baseline to 77% at follow-up.

**Table 2:** Acts of Sexual Bullying or Gender Harassment (SBGH) ever witnessed by boys who were unmarried and 12-16 years at baseline and retained at follow-up, Kapilvastu and Rupandehi Districts, Nepal, 2019-2022, N = 1,143.

	Baseline	Follow-Up	p-value^1^
Number of acts of SBGH ever witnessed, by type and overall	M (SE) α^2^	M (SE) α^2^	
Non-contact acts [0–7]	1.59 (0.07) 0.68	1.59 (0.06) 0.59	0.93
Contact acts [0–7]	0.87 (0.05) 0.71	0.69 (0.07) 0.63	<0.01
Non-contact or contact acts [0–14]	2.46 (0.10) 0.79	2.28 (0.11) 0.75	0.06
**Prevalence of** non-contact **acts of SBGH ever witnessed, by type and overall**	**% Yes**	**% Yes**	
Making sexual comments, jokes, movements, or looks at any girl	50.48	52.32	0.36
Spreading sexual rumors about a girl	31.58	24.41	<0.01
Calling a girl “fag,” “dyke,” “lezzie,” or “queer”	21.35	16.54	<0.01
Flashing or “mooning” a girl	8.22	4.72	<0.01
Spying on a girl as they dressed or showered	9.62	4.81	<0.01
Showing, giving, or sending a girl sexual pictures, photographs, messages, or notes	12.42	6.91	<0.01
Writing sexual messages or graffiti … about a girl	25.02	49.52	<0.01
Any non-contact acts	63.78	77.43	<0.01
**Prevalence of** contact **acts of SBGH ever witnessed**	**% Yes**	**% Yes**	
Brushing up against a girl in a sexual way on purpose	29.13	21.26	<0.01
Pulling at a girl’s clothing in a sexual way	10.50	11.72	0.36
Blocking a girl’s way or cornering her in a sexual way	12.77	9.54	0.01
Forcing a girl to do something sexual other than kissing	12.16	8.05	<0.01
Forcing a girl to kiss	7.44	5.34	0.04
Touching, grabbing, or pinching a girl in a sexual way	6.12	10.06	<0.01
Pulling a girl’s clothing off or down	9.01	2.62	<0.01
Any contact acts of SBGH ever witnessed	42.26	37.71	0.02
Any non-contact or contact acts of SBGH ever witnessed	67.89	82.68	<0.01

^1^Based on paired t-test of difference in proportion or mean.

^2^Cronbach’s alpha reliability.

ⴕp < 0.1;

*p < 0.05;

**p < 0.01.

At baseline, boys frequently reported ever witnessing another boy or peer engage in *contact* forms of bullying or harassment of a girl; however, boys generally reported ever witnessing these acts less often than non-contact forms of bullying or harassment (Table 2). The baseline reported prevalence of ever witnessing any of seven acts of contact bullying or harassment was 42%, and the reported prevalence of ever witnessing specific acts ranged from 6% for “touching, grabbing, or pinching a girl in a sexual way” to 29% for “brushing up against a girl in a sexual way on purpose.” From baseline to follow-up, the reported prevalence of ever witnessing a boy or male peer “touching, grabbing, or pinching a girl in a sexual way” increased modestly (from 6% to 10%) and the reported prevalence of “pulling at a girl’s clothing in a sexual way” remained stable (at 10% and 12%); however, the reported prevalence of ever witnessing other listed acts of contact bullying or harassment *implausibly declined* (Table 2). As a result, the overall reported prevalence of ever witnessing another boy or peer engaging in any contact form of bullying or harassment of a girl *implausibly declined*, from 42% at baseline to 38% at follow-up. Because of the marked increase in ever witnessing another boy or male peer engage in “writing sexual messages or graffiti … about a girl,” the overall prevalence of ever witnessing any *non-contact or contact* act of bullying or harassment of a girl increased, from 68% at baseline to 83% at follow-up.

### Ever witnessing sexual bullying or gender harassment by study arm

Table 3 compares mean counts and prevalences of boys’ reports of ever witnessing another boy or male peer engaging in acts of non-contact or contact forms of bullying and harassment of a girl, overall and by study arm at baseline and follow-up. (For act-specific comparisons, see S1 Table.) The mean counts of acts of *non-contact* bullying or harassment of a girl ever witnessed at baseline and follow-up, respectively, were: 1.6 and 1.8 in the control group, 1.4 and 1.5 in TPP, and 1.7 and 1.4 in TPP + . The mean counts of acts of *contact* bullying or harassment of a girl ever witnessed at baseline and follow-up, respectively, were: 0.9 and 0.7 in the control group; 0.9 and 0.8 in TPP; and 0.9 and 0.6 in TPP + . Overall, the mean counts of acts of *non-contact or contact* bullying or harassment of a girl ever witnessed at baseline and follow-up, respectively, were: 2.5 and 2.5 in the control group; 2.3 and 2.3 in TPP; and 2.6 and 2.1 in TPP + .

**Table 3:** Means (SE) and prevalences for ever witnessing contact, non-contact, or any (Contact or Non-Contact) Sexual Bullying or Gender Harassment (SBGH) of girls, as reported by unmarried boys 12-16 years at baseline who were retained at follow-up, overall and by study arm, Kapilvastu and Rupandehi Districts, Nepal, 2019-2022.

	Baseline				Follow-Up			
	Control (n = 391)	TPP (n = 390)	TPP Plus (n = 362)	Overall (n = 1,143)	Control (n = 391)	TPP (n = 390)	TPP Plus (n = 362)	Overall (n = 1,143)
**Panel A: Mean and Standard Error (SE) for the Number of Acts of SBGH Ever Witnessed, by Type, Overall and by Study Arm**								
**# of Acts**	**M (SE)**	**M (SE)**	**M (SE)**	**M (SE)**	**M (SE)**	**M (SE)**	**M (SE)**	**M (SE)**
Non-contact	1.64 (0.11)	1.41 (0.11)	1.72 (0.13)	1.59 (0.07)	1.79 (0.10)	1.54 (0.12)	1.44 (0.10)	1.59 (0.06)
Contact	0.86 (0.06)	0.89 (0.11)	0.87 (0.09)	0.87 (0.05)	0.68 (0.12)	0.75 (0.12)	0.62 (0.10)	0.69 (0.07)
Contact or non-contact	2.50 (0.13)	2.30 (0.20)	2.58 (0.18)	2.46 (0.10)	2.47 (0.20)	2.29 (0.21)	2.06 (0.16)	2.28 (0.11)
**Panel B: Prevalence of Any SBGH Ever Witnessed, by Type, Overall and by Study Arm**								
**Prevalence**	**% Yes**	**% Yes**	**% Yes**	**%Yes**	**% Yes**	**% Yes**	**% Yes**	**%Yes**
Non-Contact	65.98	58.21	67.40	63.78	79.03	75.90	77.35	77.43
Contact	42.97	38.72	45.30	42.26	36.32	37.95	38.95	37.71
Contact or non-contact	70.08	63.08	70.72	67.89	81.84	82.82	83.43	82.68

Also shown in Table 3, the following percentages of boys reported ever witnessing any *non-contact* bullying or harassment of a girl at baseline and follow-up, respectively: 66% and 79% in the control group, 58% and 76% in TPP, and 67% and 77% in TPP + . The following percentages of boys reported ever witnessing any *contact* bullying or harassment of a girl at baseline and follow-up, respectively: 43% and 36% in the control group, 39% and 38% in TPP, and 45% and 39% in TPP + . Overall, the following percentages of boys reported ever witnessing any *non-contact or contact* acts of bullying or harassment of a girl at baseline and follow-up, respectively: 70% and 82% in the control group, 63% and 83% in TPP, and 71% and 83% in TPP + .

### Results from difference-in-difference models

Table 4 presents the results for the unadjusted and adjusted difference-in-difference (DID) analyses of outcomes related to non-contact bullying or harassment ever witnessed, contact bullying or harassment ever witnessed, and contact or non-contact bullying or harassment ever witnessed. (For act-specific DID models, see S2 Table.) In unadjusted models, for TPP+ versus the control group, regression-based DID models showed significant negative program effects for ever witnessing contact or non-contact bullying or harassment (β_5_ coef. for G_3_ = -0.50, p < .10) and for witnessing non-contact bullying or harassment (β_5_ coef. for G_3_ = -0.43, p < .05). In adjusted models that controlled for individual and community factors, for TPP+ versus the control group, regression-based DID models showed significant negative program effects for ever witnessing non-contact bullying or harassment (β_5_ coef. for G_3_ = -0.42, p < .05). For TPP versus the control group, regression-based DID models showed no significant program effects. Thus, compared to boys in the control group, boys assigned to the TPP+ group had lower adjusted odds of reporting to have ever witnessed another boy or male peer engage in non-contact forms of bullying or harassment of a girl.

**Table 4:** Results from unadjusted and adjusted difference-in-difference models for the effects of assignment to the CARE Tipping Point Program (TPP) or CARE Tipping Point Plus Program (TPP+) on the number of acts of sexual bullying or gender harassment of girls ever witnessed, as reported by unmarried boys 12-16 years at baseline who were retained at follow-up, Kapilvastu and Rupandehi Districts, Nepal, 2019-2022 (N = 1,143).

	Ever witness Contact or Non-Contact SBGH		Ever witness Contact SBGH		Ever witness Non-contact SBGH	
	Est.	95% CI	Est.	95% CI	Est.	95% CI
**Panel A: Unadjusted Models**						
TPP (G_2_)	0.01	-0.78, 0.80	0.04	-0.41, 0.49	-0.03	-0.46, 0.40
TPP+ (G_3_)	**-0.50** ^ⴕ^	**-1.11, 0.10**	-0.07	-0.45, 0.31	-0.43*	**-0.79, -0.07**
**Panel B: Adjusted Models** ^1^						
TPP (G_2_)	0.01	-0.78,0.81	0.03	-0.42,0.48	-0.02	-0.45, 0.41
TPP+ (G_3_)	-0.50	-1.11, 0.11	-0.08	-0.46,0.30	-0.42*	**-0.79, -0.06**

ⴕp < 0.10;

*p < 0.05;

**p < 0.01.

^1^Models adjusted for age in years, read and/or write, grades completed, still attending school, received vocational training, household religion, caste, Household PPI, male head primary occupation, other (non-TPI) empowerment organizations attended, proportion of households from an advantaged caste, proportion of households being Muslim, average household PPI score, mean grades of schooling completed for women 25 years or older, and the gender gap in mean grades completed for adults 25 years or older (men’s mean grades – women’s mean grades)

## Discussion

### Summary and interpretation

This paper leveraged data from a large, community-based cluster-randomized controlled trial that originally was designed to test the effects of the CARE Tipping Point Program (TPP) and Tipping Point Plus Program (TPP+) on the primary outcome of CEFM and secondary agency-related outcomes among adolescent girls, which are reported elsewhere [12]. One component of the CARE TPP and TPP + was designed to engage men and boys as structured allies in girl-centered efforts to change the harmful community-level gender norms that underlie CEFM and VAWG, more generally. Thus, the focal secondary outcome here was reports by adolescent male participants of ever witnessing other boys or male peers commit acts of non-contact and contact sexual bullying or gendered harassment of girls. Findings have important implications for understanding whether girl-centered programs that engage men and boys as structured allies can have significant favorable effects on the ‘day-to-day’ forms of violence that girls may experience.

Based on our findings, boys reported high baseline levels of ever witnessing non-contact (63.8%), contact (42.3%), or any (67.9%) acts of sexual bullying or gender harassment against a girl by male peers. Compared to self-reports of bullying perpetration by boys in other South Asian settings, estimated levels of ever witnessing bullying by male peers in the present sample were of a similar magnitude. For example, in school-based studies, 16% to 85% of boys 12–14 years in Pakistan and 62% of boys 12–20 years in Nepal have reported bullying perpetration [29]. While reports of witnessing the behavior male peers and self-reports of perpetration are not directly comparable, the similarities in our estimates especially with those from other studies in Nepal suggest that asking about witnessing sexual bullying and gender harassment can yield reasonable estimates of prevalence.

Boys also reported significant changes from baseline to follow-up in ever witnessing non-contact and contact forms of sexual bullying and harassment toward girls by other boys. The direction of changes in specific acts were not uniform, and some changes were implausible, given the cumulative nature of the outcome under study. At baseline, 64% of boys reported to have ever witnessed any of seven acts of non-contact sexual bullying or gender harassment, and this overall reported prevalence *increased* to 77% at follow-up. This overall increase was due largely to a sharp increase in boys’ reports of ever witnessing other boys “writing sexual messages … about a girl.” Otherwise, the reported prevalence of ever witnessing one act remained plausibly stable, and reports for all other acts *implausibly* declined.

The distinction between sharp increases in “writing sexual messages … about a girl” and implausible declines in reports of *ever witnessing* other forms of non-contact sexual bullying and gender harassment has several potential explanations. First, the social distancing protocols imposed during COVID-19 may have resulted in a shift from more direct forms of non-contact sexual bullying and gender harassment to more indirect or anonymous forms, such as “writing sexual messages … about a girl.” While this shift could explain the sharp increase in “writing sexual messages” during the project period, it would not explain the *implausible declines* in reports of *ever* witnessing more direct forms of non-contact sexual bullying and gender harassment. Second, increased access to mobile phones, the internet, and social media may have contributed to a more general increase in writing sexual messages, whether online or by other written means, as the question was worded (“writing sexual messages or graffiti (e.g., on bathroom walls, in locker rooms, in a note or book) about a girl”). Bullies may have found it preferable to write sexual messages, which may have appeared to provide greater anonymity and reduced risk of consequences. The indirect spreading of sexual rumors about a girl is a well-identified and prevalent form of sexual bullying and gender harassment [67]. Again, this shift would explain the sharp increase in reports of ever witnessing a male peer “writing sexual messages,” but it would not explain implausible declines in reports of ever witnessing other, direct acts of non-contact sexual bullying and gender harassment. Third, boys may have misunderstood the item at baseline, and upon learning more about this behavior during the study period, reported much higher prevalences at follow-up. While this explanation also is plausible, the statement included specific clarifications and was cognitively tested before the baseline interview. Finally, to explain the implausible trends in ever witnessing these other acts, participants in TPP + may have learned through the programming that these direct acts were more socially undesirable and carried with them substantial consequences. As a result, at follow-up, male participants in TPP + may have concealed prior reports of having ever witnessed direct acts of non-contact sexual bullying and gender harassment that they had learned from TPP + to be undesirable.

Further analyses of specific acts of non-contact sexual bullying and gender harassment corroborate this third explanation. Specifically, in unadjusted (S1 Table) and adjusted (S2 Table) analyses, boys in the TPP+ group and in the control group reported *similar plausible increases* in ever witnessing other boys “write sexual messages.” However, while boys in the control group had plausibly stable or increased likelihoods of reporting to have ever witnessed another boy call a girl derogatory names or show a girl sexual images or notes, boys in the TPP+ group had implausible declines in the likelihoods of reporting to have ever witnessed these acts.

The results for reports of ever witnessing contact forms of sexual bullying or gender harassment further corroborate this third explanation. At baseline, a lower but substantial percentage (42%) of boys had reported to have ever witnessed any of seven acts of contact sexual bullying or gender harassment of a girl, and this overall prevalence declined to 38% at follow-up (S1 Table). The prevalence of ever witnessing specific acts ranged widely at baseline (from 6% to 29%), and except for one specific act (“pulling at a girls’ clothing in a sexual way”), all these reported forms of sexual bullying and gender harassment *implausibly declined* by follow-up (to 3% and 22%). These declines, however, were uniform across treatment and control groups and across specific acts, such that adjusted DID models do not demonstrate TPP or TPP+ impacts relative to the control condition on reports of ever witnessing contact forms of sexual bullying or gender harassment (S2 Table). Known programming in the control groups may have contributed to these similar implausible trends [12].

Thus, significant declines in boys’ accounts of ever witnessing non-contact sexual bullying and harassment of girls by their male peers may have been attributable to greater concealment of undesirable behavior among boys who had been exposed to the Tipping Point Plus Program (TPP+). As noted in the background section and elsewhere [12,62,68], the TPP+ package was distinctive in its efforts to engage community leaders to support change in repressive community-level norms about gender and girls’ sexuality and in its efforts to support girl-led community events to change gender norms. Although the COVID-19 pandemic diminished the planned frequency, scope, and intensity of community-level engagement activities in clusters assigned to TPP + , sustained TPP+ programming may have been sufficiently distinct from the control condition to have changed boys’ reports more of *ever* witnessing non-contact forms of sexual bullying and gender harassment of a girl by their male peers. Thus, engaging community leaders in the TPP+ group may have spurred greater awareness among boys in this group of the consequences of some forms of sexual bullying and gender harassment against girls, and a desire among them to protect their male peers by reversing prior reports of ever having witnessed such acts. One also cannot rule out the possibility that boys in the TPP+ group were more likely than boys in the control group to “correct” their baseline overreports of ever witnessing certain non-contact forms of sexual bullying and gender harassment; however, the behaviorally based nature of these questions reduces the plausibility of this explanation.

### Limitations and strengths of the analysis

Some limitations of this analysis warrant discussion. First, despite the randomization of ward clusters to study arms, the samples of adolescent boys in the TPP and TPP+ study arms may have differed from boys in their communities because of the voluntariness of participation in CARE programming. As a result, inferences from this analysis may not be attributable to the population of 12–16 year-old boys in the communities from which participants were drawn. A detailed discussion of this limitation is available in the published study protocol, as are methods to assess the balance of baseline characteristics across TPP, TPP + , and control arms [62]. Following the published protocol, this balance check was applied in the primary impact assessment of CEFM among girls [12] and in the present analysis of witnessing SBGH among boys (Tables 1 and 3; S1 Table). Based on these balance checks and theory, potential individual- and community-level confounders of the estimated impacts of TPP and TPP+ were controlled in prior analyses [12] and in those presented here (Table 4; S2 Table). Thus, while heterogeneity in unobserved confounders cannot be ruled out, a comparison of DID models that are unadjusted and adjusted for a range of confounders reveal that the within-sample treatment effects are reasonably robust.

Second, Tipping Point programming was well underway before the onset of the COVID-19 pandemic. CARE USA and CARE Nepal followed institutional and national guidelines for COVID-19 risk-mitigation strategies and paused the implementation of programming for five months, from March through July of 2020. In addition, unplanned modifications to program components occurred after program implementation had resumed. Particularly relevant modifications included the truncation of large community gatherings that were part of the TPP+ package. These community gatherings—designed to bring norm-shifting behaviors to open, public forums for community reflections—were critical to the TPP+ theory of change. This unplanned hiatus in TPP and TPP+ programming and modifications, especially to TPP + , likely reduced the extent of program participation, retained learning among participants, and the nature and magnitude of behavioral change that may have been underway among participants before the onset of the pandemic. The truncation of large community gatherings also may mean that the effects observed in this analysis are likely to be lower than the effects that would have been observed if large gatherings had been possible. Future programmatic efforts that involve social-movement building might consider other, technology-based ways of mobilizing large groups of adolescent girls, adolescent boys, and adults at the community level.

A third notable limitation of the analysis was that reports of ever witnessing any of the different acts assessed appear to have been subject to social desirability bias and “frequency illusion.” An even greater threat to inferences about program impact would be differential changes across study arms in social desirability bias and the capacity or willingness of boys to recall and to report ever witnessing acts of sexual bullying and gender harassment of a girl by another boy or male peer [69]. The research team attempted to mitigate social desirability bias generally by asking questions that utilized anonymous peer reporting [64]. Specifically, boys were not asked to report on their own sexual bullying or gender harassment behavior, nor were they asked to name the peers that they reported to have ever witnessed enacting these behaviors. This form of asking about sensitive behaviors was expected to improve disclosure and to reduce the effects of socially desirable responding [69]. While declines in reporting to have ever witnessed most acts appear to have been uniform across treatment and control groups (S1 and S2 Tables), the results were suggestive of differential social desirability bias or frequency illusion for reports of witnessing some acts of SBGH across study arms.

A fourth limitation was the lower reliability of the subscales for non-contact and contact forms of sexual bullying and gender harassment at endline (Table 2). One explanation for this lower reliability may have been the difficulty of translating some of the forms of bullying from English into Nepali and Awadhi. For some terms, such as “fag,” “dyke,” “lezzie,” or “queer,” there were no direct translations into Nepali or Awadhi. Still, attempts were made to find equivalent terms, and the terms used in Nepali and Awadhi were the same. Despite this limitation, both sub-scales had adequate or very near adequate reliability at baseline, and the combined scale had adequate reliability at baseline and follow-up. Also, despite the lower reliability of the scale for ever witnessing acts of non-contact sexual bullying and gender harassment, the adjusted “impact” of TPP + on this outcome remained significant.

A fifth limitation of the study was the absence of information on contact/non-contact sexual bullying and gender harassment from parents or other community members or administrative reports. Despite this limitation, research on violence generally recommends not collecting data on violent behavior from multiple members of the same household to avoid breeches of confidentiality and potential backlash against the perpetrator or victim. Also, parents may not be able to report accurately about their sons’ SBGH behavior against girls, which would be more likely to occur outside the home in venues (e.g., at school) where parents would not have the opportunity to observe their sons’ behavior. Also, administrative records on acts of sexual bullying and gender harassment are notoriously poor, given the stigma and backlash that victims who report may experience. Until institutional environments are more conductive to victim reporting of experiences of SBGH, direct self-reports of victimization and perpetration in anonymous or confidential surveys may be the preferred avenue for data collection.

A final limitation of the analysis was that the sample size of boys was not powered a priori to test for modifications of the impacts of TPP and TPP + by age on reports of ever witnessing sexual bullying and gender harassment of girls by other male peers. It remains possible that programmatic effects differed between younger and older boys in ways that are masked by this age-aggregated analysis. To explore this possibility, we conducted an ancillary analysis of the impacts of TPP and TPP + on ever witnessing sexual bullying or gender harassment, stratified by age, comparing the results for 12–14 year-old boys versus 15–16 year-old boys (S3 and S4 Tables). Qualitatively, among the older boys compared to the younger boys, reports of ever witnessing sexual bullying or gender harassment were higher at baseline and implausibly declined more from baseline to follow-up (available on request), although not significantly so based on overlapping confidence intervals for the treatment effect. Thus, it is possible that differential social desirability bias was more prominent among the older boys in this sample. Modification of the impacts of TPP and TPP + by other social strata, such as caste or religion, also are plausible; however, again, the main analyses were now powered a priori to test for effect modification by caste and religion, and a minority of the study population was non-Hindu (11.5%) or from a disadvantaged caste (17.5%) (Table 1), suggesting caution with further testing for effect modification in this sample.

### Implications for research and practice engaging boys in prevention

The findings from this study have important implications for future research on programs designed to engage boys as structured allies to prevent these and other forms of VAWG. First, future impact assessments of the CARE TPP and TPP+ packages (in their originally intended forms) should be designed to test the unique effects of the program components that were designed to engage men and boys to understand the incremental cost effectiveness of these program components as an explicit anti-VAWG strategy. Second, future impact assessments of the CARE TPP and TPP+ packages should be powered to test for age-modification of program impacts on the outcomes presented in this paper. Specifically, understanding potential differences in whether and to what extent the CARE TPP and TPP+ packages engaged younger versus older adolescent boys could provide important insights about the need to tailor program content and implementation strategies to be developmentally appropriate for maximum impact. Future impact assessments also should test for effect modification by religion and caste to assess whether and to what extent the CARE TPP and TPP+ packages equally engage boys from different social strata.

Third, to address the potential emergence of greater reporting bias in treatment groups associated with an unwillingness to “call out” undesirable behavior among peers, future impact assessments of boys’ engagement programs should include a measure of social desirability bias and direct assessments of changes in their personal motivation, self-efficacy, and behavior related to engagement in anti-VAWG efforts. The latter assessments should include validated measures of motivation, self-efficacy, and proactive (non-incident-based) and reactive (incident-based) intervening behavior to assess whether these stages of change in boys’ engagement significantly increase in the treatment group. Measuring these dimensions of bystander engagement is more common in bystander intervention studies to prevent sexual violence [70,71]; however, models and measurement tools to assess the stages of bystander behavioral change also exist for sexual bullying and gender harassment [72]. Such measures may capture attitudes about or extent of motivation to intervene to prevent sexual bullying and gender harassment; proactive efforts to enhance personal knowledge about sexual bullying and gender harassment as a problem in one’s school or community; specific efforts to take part in anti-sexual bullying and gender harassment groups or organizations; extent of confidence to intervene effectively when witnessing specific acts of sexual bullying or gender harassment of a girl by a male peer; and specific efforts to intervene behaviorally when witnessing an incident of sexual bullying and gender harassment of a girl by a male peer. Such measures would capture more directly the stages of change in boys’ anti-VAWG efforts and would help target programmatic efforts to address identified barriers to engagement. Rigorous psychometric testing of these measures across study arm and survey wave [73] may help researchers to identify empirical reasons for the implausible declines in ever witnessing sexual bullying and gender harassment, reported here.

Fourth, future intervention studies of program components to assess boys’ allyship to prevent VAWG might include surveys of girls to understand their reports of SBGH victimization. Self-reports of girls’ victimization would allow the researcher to see if boys’ reports of their behavior align with girls’ reports of their experiences. A key consideration for this kind of study design would be to ensure that the clusters in which girls and boys are sampled capture peer social networks that involve interactions between the girls who are surveyed and the boys who receive the intervention. The ethics of this study design would need to be considered carefully, given the risk of backlash against the girls who may report experiences of SBGH. Surveys of perpetration and victimization, for example, may need to be conducted in separate households, and adverse events may need to be monitored carefully.

Finally, impact assessments that involve repeated and extended post-treatment follow-up would help to understand the sustainability of boys’ engagement in anti-VAWG efforts after program implementation has ceased. Repeated follow-ups also could offer insights about the nature, timing, and frequency of ‘booster’ support that that might needed to ensure the positive and sustained engagement of boys in anti-VAWG efforts.

Findings from this study also have important implications for programming to engage men and boys as structured allies in anti-VAWG efforts. First, tailored programming for younger and older adolescent boys could be implemented in age-disaggregated groups to ensure that the content of programming and its delivery are developmentally appropriate. The advantages of age-disaggregated programming and groups also should be weighed against the benefits of having younger and older boys in the same groups so older boys may serve as role models for allyship and positive behavioral change. Second, age-appropriate programming could be developed to support prosocial bystander behavior among boys when they observe acts of sexual bullying and gender harassment against girls by their male peers [17,72]. Such programming would enable boys not only to recognize such acts, but also to develop the motivation, confidence, and behavioral strategies to intervene when such acts are observed. The cultivation of prosocial bystander behavior among boys in early- to mid- adolescence could accelerate normative changes in how girls are viewed and treated that carry forward into young adulthood and beyond. Such changes, in turn, may foster more favorable school climates that support the retention and academic achievements of girls in secondary school.

Policy makers also may need to weigh the cost effectiveness of various programs designed to increase boys’ allyship and engagement in anti-VAWG efforts. Understanding what works is as important as understanding what does *not* work to ensure that resources are allocated efficiently. Based on the evidence from the present study and other interventions that work to engage young men in anti-VAWG efforts [74,75], social-norms-based programs that help boys to understand the full range of acts that constitute SBGH, address misperceptions about SBGH as ubiquitous and socially expected behavior, present proactive and reactive bystander intervention as normative behavior instead, cultivate empathy for the victims of SBGH, and strengthen self-efficacy [74,75] may enable boys to intervene effectively with other boys and to change their own behavior in a programmatic environment that supports change.

## Conclusion

From baseline to follow-up, compared to boys in the control group, boys who participated in the CARE Tipping Point Plus Program implausibly reported ever witnessing fewer acts of non-contact sexual bullying and gender harassment against girls by other boys in Kapivastu and Rupandehi districts of Nepal. Revisions to study designs, measures, and engagement programming that emphasize proactive and reactive bystander behavior as normative may be needed to realize the true allyship and engagement of boys in anti-VAWG efforts.

## Supporting information

S1 Table Means (SE) and Prevalences for Ever Witnessing Contact, Non-Contact, or Any (Contact or Non-Contact) Sexual Bullying or Gender Harassment of Girls, as Reported by Adolescent Boys 12–16 Years Present at Baseline and Follow-up, Overall and by Study Arm, Kapilvastu and Rupandehi Districts, Nepal, 2019–2022. (DOCX)

S2 Table Results from Unadjusted and Adjusted Difference-in-Difference Models for the Effects of Assignment to the CARE Tipping Point Program (TPP) or CARE Tipping Point Plus Program (TPP+) on Ever Witnessing Sexual Bullying or Gender Harassment of Girls, as Reported by Adolescent Boys who were Unmarried and 12–16 Years Old at Baseline and Present at Follow-up, Kapilvastu and Rupandehi Districts, Nepal, 2019–2022 (N = 1,143). (DOCX)

S3 Table Results from Unadjusted and Adjusted Difference-in-Difference Models for the Effects of Assignment to the CARE Tipping Point Program (TPP) or CARE Tipping Point Plus Program (TPP+) on the Number of Acts of Sexual Bullying or Gender Harassment of Girls Ever Witnessed, as Reported by Unmarried Boys 12–14 Years at Baseline who were Retained at Follow-up, Kapilvastu and Rupandehi Districts, Nepal, 2019–2022 (N = 773). (DOCX)

S4 Table Results from Unadjusted and Adjusted Difference-in-Difference Models for the Effects of Assignment to the CARE Tipping Point Program (TPP) or CARE Tipping Point Plus Program (TPP+) on the Number of Acts of Sexual Bullying or Gender Harassment of Girls Ever Witnessed, as Reported by Unmarried Boys 15–16 Years at Baseline who were Retained at Follow-up, Kapilvastu and Rupandehi Districts, Nepal, 2019–2022 (N = 410). (DOCX)

S1 File Dataset. (DTA)
