# Supplementary material for: Engaging boys as “Structured Allies” to prevent gender-based violence against girls: Results from the CARE Tipping Point Initiative in Nepal
Source: PLoS One. 2025 May 15;20(5):e0320014. doi: 10.1371/journal.pone.0320014 (PMC12080825; doi:10.1371/journal.pone.0320014)
Supplement: S1 Table — (DOCX) [file pone.0320014.s001.docx]

| **S1 Table: Means (SE) and Prevalences for Ever Witnessing Contact, Non-Contact, or Any (Contact or Non-Contact) Sexual Bullying or Gender Harassment of Girls, as Reported by Adolescent Boys 12-16 Years Present at Baseline and Follow-up, Overall and by Study Arm, Kapilvastu and Rupandehi Districts, Nepal, 2019-2022** | | | | | | | | |
| --- | --- | --- | --- | --- | --- | --- | --- | --- |
|  | **Baseline** | | | | **Follow-Up** | | | |
|  | **Control**  **(n=391)** | **TPP**  **(n=390)** | **TPP+**  **(n=362)** | **Overall**  **(n=1,143)** | **Control**  **(n=391)** | **TPP**  **(n=390)** | **TPP+**  **(n=362)** | **Overall**  **(n=1,143)** |
| **Panel A: Mean and standard error (SE) for the number of acts of sexual bullying or gender harassment ever witnessed, by type, overall and by study arm** | | | | | | | | |
| Number of acts reportedly ever witnessed | M, (SE) | M, (SE) | M, (SE) | M, (SE) | M, (SE) | M, (SE) | M, (SE) | M, (SE) |
| Non-Contact | 1.64 (0.11) | 1.41 (0.11) | 1.72 (0.13) | 1.59 (0.07) | 1.79 (0.10) | 1.54 (0.12) | 1.44 (0.10) | 1.59 (0.06) |
| Contact | 0.86 (0.06) | 0.89 (0.11) | 0.87 (0.09) | 0.87 (0.05) | 0.68 (0.12) | 0.75 (0.12) | 0.62 (0.10) | 0.69 (0.07) |
| Any Contact/Non-Contact | 2.50 (0.13) | 2.30 (0.20) | 2.58 (0.18) | 2.46 (0.10) | 2.47 (0.20) | 2.29 (0.21) | 2.06 (0.16) | 2.28 (0.11) |
| **Panel B: Prevalence of sexual bullying or gender harassment ever witnessed, by type, overall and by study arm** | | | | | | | | |
| Prevalence of acts reportedly ever witnessed | % Yes | % Yes | % Yes | %Yes | % Yes | % Yes | % Yes | %Yes |
| Non-Contact | 65.98 | 58.21 | 67.40 | 63.78 | **79.03** | 75.90 | **77.35** | 77.43 |
| Making sexual comments, jokes, movements, or looks at any girl | 53.71 | 47.18 | 50.55 | 50.48 | **53.45** | 53.33 | **50.00** | 52.32 |
| Spreading sexual rumors about a girl | 33.25 | 25.64 | 36.19 | 31.58 | 29.41 | 22.56 | 20.99 | 24.41 |
| **Calling a girl “fag,” “dyke,” “lezzie,” or “queer”** | **20.46** | **19.49** | **24.31** | **21.35** | **23.79** | **14.36** | **11.05** | **16.54** |
| Flashing or “mooning” a girl | 7.42 | 8.21 | 9.12 | 8.22 | 4.86 | 4.87 | 4.42 | 4.72 |
| Spying on a girl as they dressed or showered | 7.16 | 10.51 | 11.33 | 9.62 | 3.84 | 6.41 | 4.14 | 4.81 |
| **Showing, giving, or sending a girl sexual pictures, photographs, messages, or notes** | **12.28** | **10.26** | **14.92** | **12.42** | **11.25** | **6.15** | **3.04** | **6.91** |
| Writing sexual messages or graffiti … about a girl | 29.67 | 20.26 | 25.14 | 25.02 | **52.43** | 46.15 | **50.00** | 49.52 |
| Contact | 42.97 | 38.72 | 45.30 | 42.26 | 36.32 | 37.95 | 38.95 | 37.71 |
| Brushing up against a girl in a sexual way on purpose | 28.64 | 25.90 | 33.15 | 29.13 | 23.27 | 21.03 | 19.34 | 21.26 |
| Pulling at a girl’s clothing in a sexual way | 11.76 | 9.23 | 10.50 | 10.50 | 9.97 | 12.05 | **13.26** | 11.72 |
| Blocking a girl’s way or cornering her in a sexual way | 11.51 | 14.62 | 12.15 | 12.77 | 9.72 | 11.28 | 7.46 | 9.54 |
| Forcing a girl to do something sexual other than kissing | 11.25 | 12.82 | 12.43 | 12.16 | 6.14 | 8.72 | 9.39 | 8.05 |
| Forcing a girl to kiss | 6.91 | 9.74 | 5.52 | 7.44 | 6.39 | 6.15 | 3.31 | 5.34 |
| Touching, grabbing, or pinching a girl in a sexual way | 5.88 | 6.92 | 5.52 | 6.12 | **10.23** | 12.56 | **7.18** | 10.06 |
| Pulling a girl’s clothing off or down | 9.97 | 9.49 | 7.46 | 9.01 | 2.56 | 3.33 | 1.93 | 2.62 |
| Any Contact/Non-Contact | 70.08 | 63.08 | 70.72 | 67.89 | 81.84 | 82.82 | **83.43** | 82.68 |
