# Supplementary material for: Engaging boys as “Structured Allies” to prevent gender-based violence against girls: Results from the CARE Tipping Point Initiative in Nepal
Source: PLoS One. 2025 May 15;20(5):e0320014. doi: 10.1371/journal.pone.0320014 (PMC12080825; doi:10.1371/journal.pone.0320014)
Supplement: S2 Table — (DOCX) [file pone.0320014.s002.docx]

| **S2 Table: Results from Unadjusted and Adjusted Difference-in-Difference Models for the Effects of Assignment to the CARE Tipping Point Program (TPP) or CARE Tipping Point Plus Program (TPP+) on Ever Witnessing Sexual Bullying or Gender Harassment of Girls, as Reported by Adolescent Boys who were Unmarried and 12-16 Years Old at Baseline and Present at Follow-up, Kapilvastu and Rupandehi Districts, Nepal, 2019-2022 (N=1,143)** | | | | |
| --- | --- | --- | --- | --- |
|  | **Unadjusted (ref: control)** | | **Adjusted (ref: control)^1^** | |
|  | **TPP** | **TPP+** | **TPP** | **TPP+** |
|  | Coef. (95% CI) | Coef. (95% CI) | Coef. (95% CI) | Coef. (95% CI) |
| **Any non-contact sexual bullying or gender harassment ever witnessed** | -0.03 (-0.46, 0.40) | **-0.43 (-0.79, -0.07)*** | -0.02 (-0.45, 0.41) | **-0.42 (-0.79, -0.06)*** |
| Making sexual comments, jokes, movements, or looks at any girl | 0.06 (-0.10, 0.22) | -0.00 (-0.15, 0.14) | 0.06 (-0.10, 0.23) | -0.00 (-0.15, 0.14) |
| Spreading sexual rumours about a girl | 0.01 (-0.13, 0.15) | -0.11 (-0.27, 0.04) | 0.01 (-0.13, 0.15) | -0.11 (-0.27, 0.04) |
| **Calling a girl “fag,” “dyke,” “lezzie,” or “queer”** | **-0.08 (-0.19, 0.02)** | **-0.17 (-0.24, -0.09)**** | **-0.08 (-0.19, 0.24)** | **-0.17 (-0.24, -0.09)**** |
| Flashing or “mooning” a girl | -0.01 (-0.06, 0.04) | -0.02 (-0.07, 0.03) | -0.01 (-0.05, 0.04) | -0.02 (-0.07, 0.03) |
| Spying on a girl as they dressed or showered | -0.01 (-0.08, 0.06) | -0.04 (-0.10, 0.02) | -0.01 (-0.08, 0.06) | -0.04 (-0.09, 0.02) |
| **Showing, giving, or sending a girl sexual pictures, photographs, messages, or notes** | **-0.03 (-0.13, 0.07)** | **-0.11 (-0.21, -0.01)*** | **-0.03 (-0.13, 0.06)** | **-0.11 (-0.21, -0.01)*** |
| Writing sexual messages or graffiti … about a girl | 0.03 (-0.10, 0.16) | 0.02 (-0.12, 0.16) | 0.03 (-0.10, 0.16) | 0.02 (-0.12, 0.16) |
| **Any contact sexual bullying or gender harassment ever witnessed** | 0.04 (-0.41, 0.49) | -0.07 (-0.45, 0.31) | 0.03 (-0.42, 0.48) | -0.08 (-0.46, 0.30 |
| Brushing up against a girl in a sexual way on purpose | 0.00 (-0.13, 0.14) | -0.08 (-0.20, 0.03) | 0.01 (-0.13, 0.14) | -0.08 (-0.20, 0.04) |
| Pulling at a girl’s clothing in a sexual way | 0.05 (-0.04, 0.13) | 0.05 (-0.03, 0.12) | 0.05 (-0.04, 0.13) | 0.05 (-0.03, 0.13) |
| Blocking a girl’s way or cornering her in a sexual way | -0.02 (-0.12, 0.09) | -0.03 (-0.12, 0.06) | -0.02 (-0.13, 0.09) | -0.03 (-0.12, 0.06) |
| Forcing a girl to do something sexual other than kissing | 0.01 (-0.09, 0.11) | 0.02 (-0.07, 0.11) | 0.01 (-0.09, 0.11) | 0.02 (-0.07, 0.11) |
| Forcing a girl to kiss | -0.03 (-0.12, 0.06) | -0.02 (-0.09, 0.05) | -0.03 (-0.12, 0.06) | -0.02 (-0.09, 0.05) |
| Touching, grabbing, or pinching a girl in a sexual way | 0.01 (-0.05, 0.08) | -0.03 (-0.10, 0.05) | 0.01 (-0.06, 0.08) | -0.03 (-0.10, 0.04) |
| Pulling a girl’s clothing off or down | 0.01 (-0.05, 0.07) | 0.02 (-0.03, 0.07) | 0.01 (-0.05, 0.07) | 0.02 (-0.04, 0.07) |
| **Any non-contact or contact sexual bullying or gender harassment ever witnessed** | 0.01 (-0.78, 0.80) | **-0.50 (-1.11, 0.10)^ⴕ^** | 0.01 (-0.78, 0.81) | -0.50 (-1.11, 0.11) |
| ⴕ p <0.10; * p<0.05; **p<0.01.  ^1^ Models adjusted for age in years, read and/or write, grades completed, still attending school, received vocational training, household religion, caste, Household PPI, male head primary occupation, other (non-TPI) empowerment organizations attended, proportion of households from an advantaged caste, proportion of households being Muslim, average household PPI score, mean grades of schooling completed for women 25 years or older, and the gender gap in mean grades completed for adults 25 years or older (men’s mean grades – women’s mean grades) | | | | |
