# Supplementary material for: Engaging boys as “Structured Allies” to prevent gender-based violence against girls: Results from the CARE Tipping Point Initiative in Nepal
Source: PLoS One. 2025 May 15;20(5):e0320014. doi: 10.1371/journal.pone.0320014 (PMC12080825; doi:10.1371/journal.pone.0320014)
Supplement: S3 Table — (DOCX) [file pone.0320014.s003.docx]

| **S3 Table: Results from Unadjusted and Adjusted Difference-in-Difference Models for the Effects of Assignment to the CARE Tipping Point Program (TPP) or CARE Tipping Point Plus Program (TPP+) on the Number of Acts of Sexual Bullying or Gender Harassment of Girls Ever Witnessed, as Reported by Unmarried Boys 12-14 Years at Baseline who were Retained at Follow-up, Kapilvastu and Rupandehi Districts, Nepal, 2019-2022 (N=773)** | | | | | | | |
| --- | --- | --- | --- | --- | --- | --- | --- |
|  | Ever Witnessed Contact or Non-Contact | | Ever Witnessed Contact | | Ever Witnessed Non-Contact | |  |
|  | Est. | 95% CI | Est. | 95% CI | Est. | 95% CI |  |
| **Panel A: Unadjusted Models^1^** | | | | | | | |
| TPP | -0.40 | -1.34, 0.54 | -0.17 | -0.67, 0.32 | -0.23 | -0.76, 0.30 |  |
| TPP+ | -0.55 | -1.40, 0.29 | -0.14 | -0.61, 0.34 | **-0.42** ^ⴕ^ | **-0.93, 0.09** |  |
| **Panel B: Adjusted Models^2^** | | | | | | | |
| TPP | -0.40 | -1.35, 0.54 | -0.17 | -0.67, 0.32 | -0.23 | -0.76, 0.30 |  |
| TPP+ | -0.56 | -1.41, 0.30 | -0.13 | -0.61, 0.35 | **-0.42** ^ⴕ^ | **-0.94, 0.09** |  |
| ⴕ p <0.10; * p<0.05; **p<0.01.  ^1^ Unadjusted Models  ^2^ Models adjusted for age in years, read and/or write, grades completed, still attending school, received vocational training, household religion, caste, Household PPI, male head primary occupation, other (non-TPI) empowerment organizations attended, proportion of households from an advantaged caste, proportion of households being Muslim, average household PPI score, mean grades of schooling completed for women 25 years or older, and the gender gap in mean grades completed for adults 25 years or older (men’s mean grades – women’s mean grades) | | | | | | | |
